# Supplementary material for: A Novel Four Mitochondrial Respiration-Related Signature for Predicting Biochemical Recurrence of Prostate Cancer
Source: J Clin Med. 2023 Jan 13;12(2):654. doi: 10.3390/jcm12020654 (PMC9866444; doi:10.3390/jcm12020654)
Supplement: Supplementary file 1 [file jcm-12-00654-s001.zip › Supplementary Table S1.pdf]

Supplementary Table S1. Clinical characteristics of PRAD patients in the TCGA cohort and GEO GSE116918

Characteristics		TCGA Total (n=400)	GEO Total (n=248)
Age (years)	>=60	233 (58.25%)	219 (88.31%)
	<60	167 (41.75%)	29 (11.69%)
Race	White	333 (83.25%)	NA
	other	54 (13.5%)	NA
	unknown	13 (3.25%)	NA
BCR	Yes	339 (84.75)	192 (77.42%)
	No	61 (15.25%)	56 (22.58%)
PSA	>=10	10 (2.50%)	198 (79.84%)
	<10	376 (94%)	50 (20.16%)
	unknown	14 (3.5%)	0
Gleason Score	>=7	361 (90.25%)	206 (83.06%)
	<7	39 (9.75%)	42 (16.94%)
Clinical_T	T1	146 (36.5%)	51(20.56%)
	T2	142 (35.5%)	76(30.65%)
	T3	38 (9.50%)	92(37.10%)
	T4	1 (0.25%)	4(1.61%)
	unknown	73 (18.25%)	25(10.08%)
laterality	bilateral	353 (88.25%)	NA
	left	17 (4.25%)	NA
	right	25 (6.25%)	NA
	unknown	5 (1.25%)	NA
Vital status	Alive	393 (98.25%)	NA
	Dead	5 (1.25%)	NA
	unknown	2 (0.50%)	NA

New tumor event after initial treatment	Yes	68 (17.00%)	NA
	No	266 (66.50%)	NA
	unknown	66 (16.50%)	NA

BCR, biochemical recurrence; PSA, prostate specific antigen; NA, not applicable.
