# Supplementary material for: A Novel Four Mitochondrial Respiration-Related Signature for Predicting Biochemical Recurrence of Prostate Cancer
Source: J Clin Med. 2023 Jan 13;12(2):654. doi: 10.3390/jcm12020654 (PMC9866444; doi:10.3390/jcm12020654)
Supplement: Supplementary file 1 [file jcm-12-00654-s001.zip › Supplementary Table S2.pdf]

**Supplementary Table S2.** Primers for qRT-PCR used in the current study.

| Primer | Sequence |
|---|---|
| APOE F | GCGGCTTGGTAAATGTGCTG |
| APOE R | AATCCCAAAAGCGACCCAGT |
| DNAH8 F | TTGTTCGTTGCCGTAATGATGT |
| DNAH8 R | ATTCTCCCTGCTTGGACTGGTT |
| EME2 F | GTGCTACTGGTGGCCTCTTGG |
| EME2 R | TGGGCTGACCCGACTGAACT |
| KIF5A F | GAGAACGATGCCGCTAAGGAT |
| KIF5A2 R | TGCCCACAATGACACTGAACT |
| internal reference H-GAPDH F | CCCATCACCATCTTCCAGG |
| internal reference H-GAPDH R | CATCACGCCACAGTTTCCC |
