# Supplementary material for: A Novel Four Mitochondrial Respiration-Related Signature for Predicting Biochemical Recurrence of Prostate Cancer
Source: J Clin Med. 2023 Jan 13;12(2):654. doi: 10.3390/jcm12020654 (PMC9866444; doi:10.3390/jcm12020654)
Supplement: Supplementary file 1 [file jcm-12-00654-s001.zip › Supplementary Table S3.pdf]

Supplementary Table S3. 13 BCR-DEGs between non-BCR and BCR samples from TCGA-PRAD.

gene	conMean	treatMean	logFC	pValue	fdr
APOE	4990.251462	8204.709677	0.717339769	0.00000414	0.000610727
CDK1	426.5614035	623.0752688	0.546653022	0.00000301	0.000591573
CPT1B	109.7309942	157.8172043	0.524283407	0.0000712	0.003819451
CXCL8	402.0526316	271.7741935	-0.564975902	0.011202579	0.106605183
CYP27A1	3020.994152	1887.043011	-0.678896085	0.006620389	0.076588818
DNAH8	1763.304094	3601.419355	1.0302843	0.031683306	0.195631603
EME2	1095.611111	1679.247312	0.616078917	0.000000513	0.00030262
FOXH1	10.14619883	14.35483871	0.500597783	0.0000091	0.000894618
HMOX1	457.8625731	647.5698925	0.500121272	0.000024	0.001575079
KIF5A	46.06140351	65.83870968	0.51537769	0.018720761	0.151304782
MTFR2	27.1871345	42.32258065	0.638503499	0.00000163	0.000480767
PAH	156.6081871	83.05376344	-0.915042187	0.01590151	0.136488999
RLBP1	0.824561404	1.365591398	0.727827038	0.041879248	0.218661561
