# Supplementary figures and images for: A Novel Four Mitochondrial Respiration-Related Signature for Predicting Biochemical Recurrence of Prostate Cancer
Source: J Clin Med. 2023 Jan 13;12(2):654. doi: 10.3390/jcm12020654 (PMC9866444; doi:10.3390/jcm12020654)

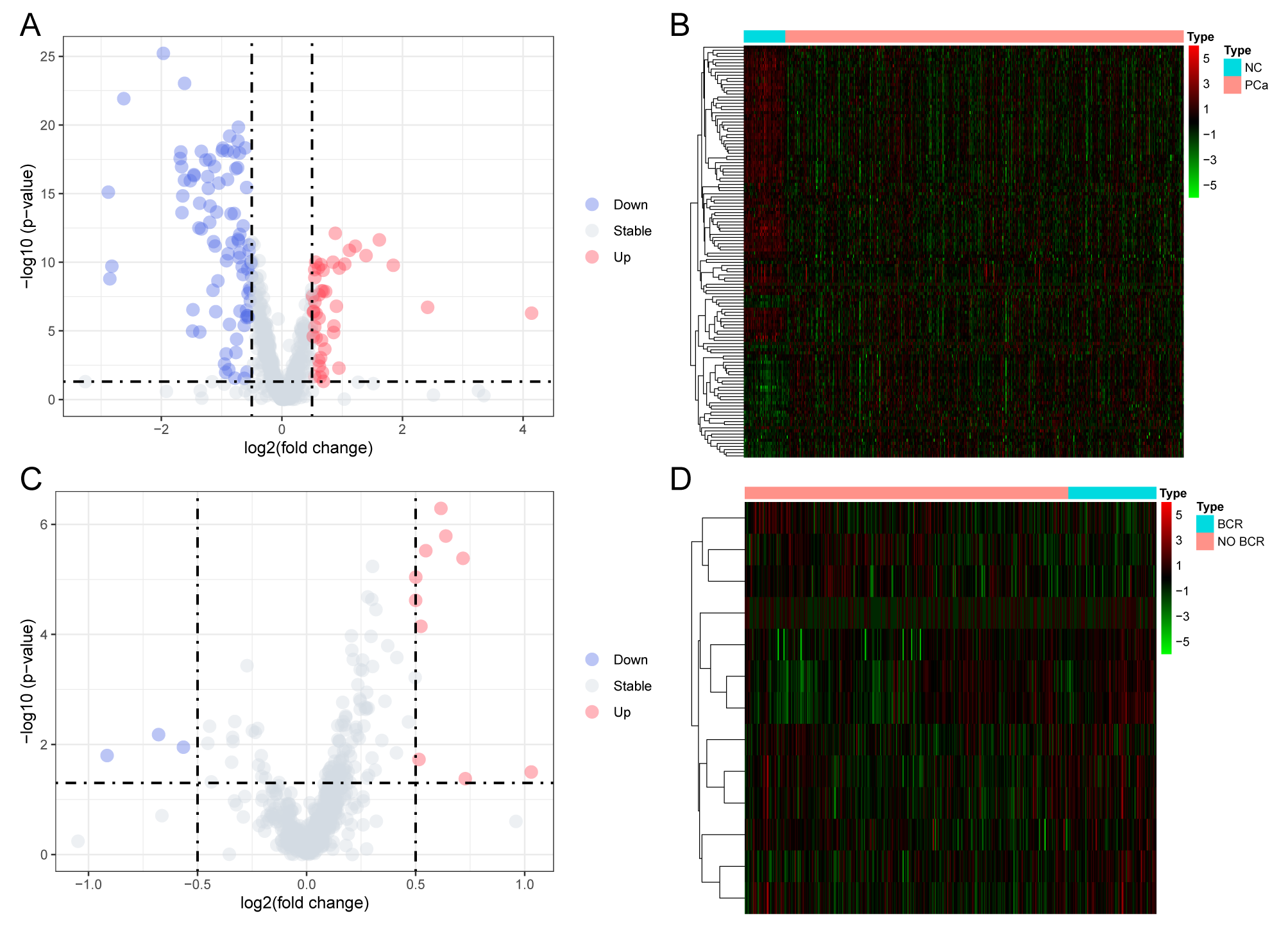

Supplement: Supplementary file 1 [file jcm-12-00654-s001.zip › Supplementary Figure 1.tif]

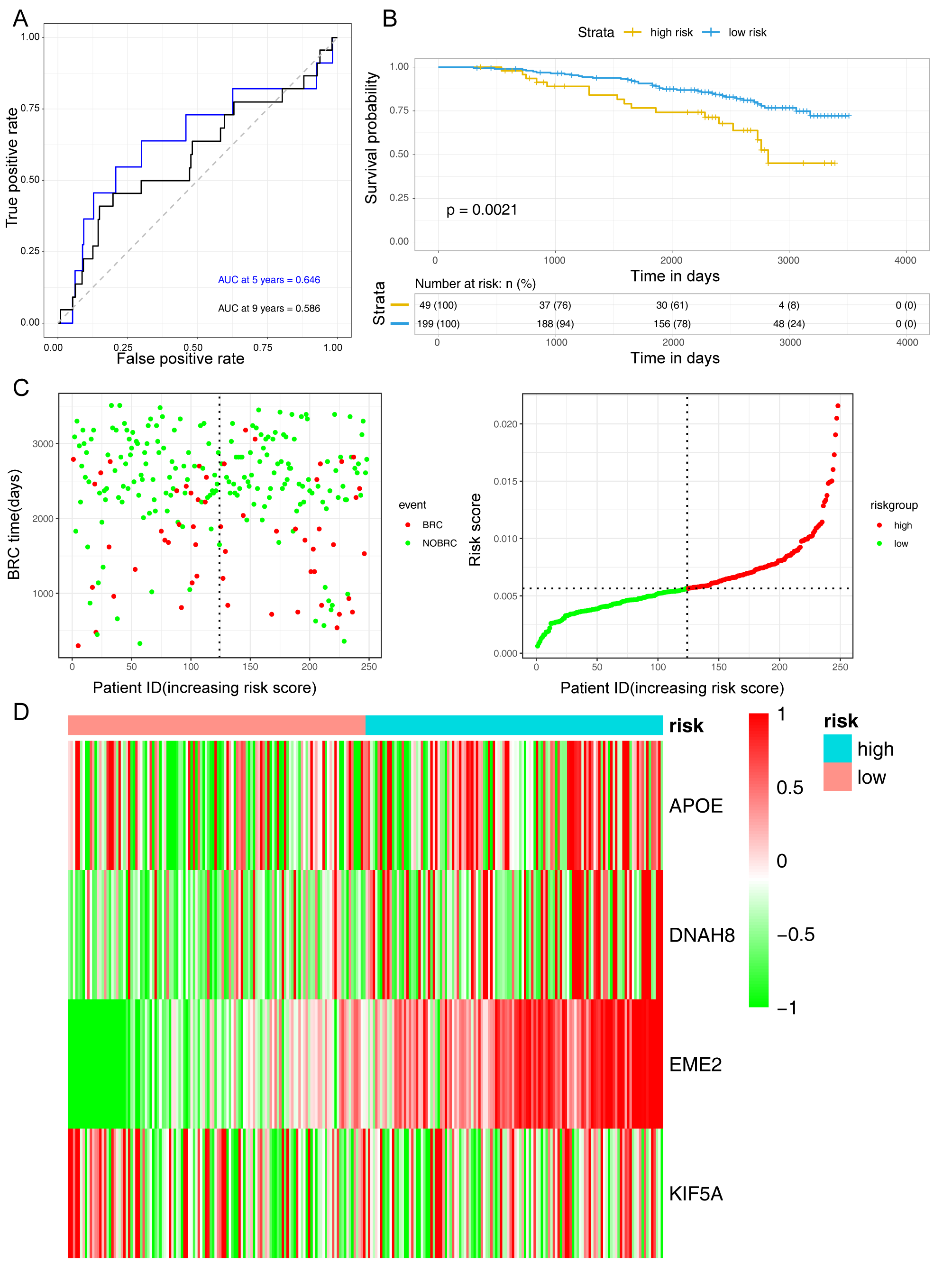

Supplement: Supplementary file 1 [file jcm-12-00654-s001.zip › Supplementary Figure 2.tif]

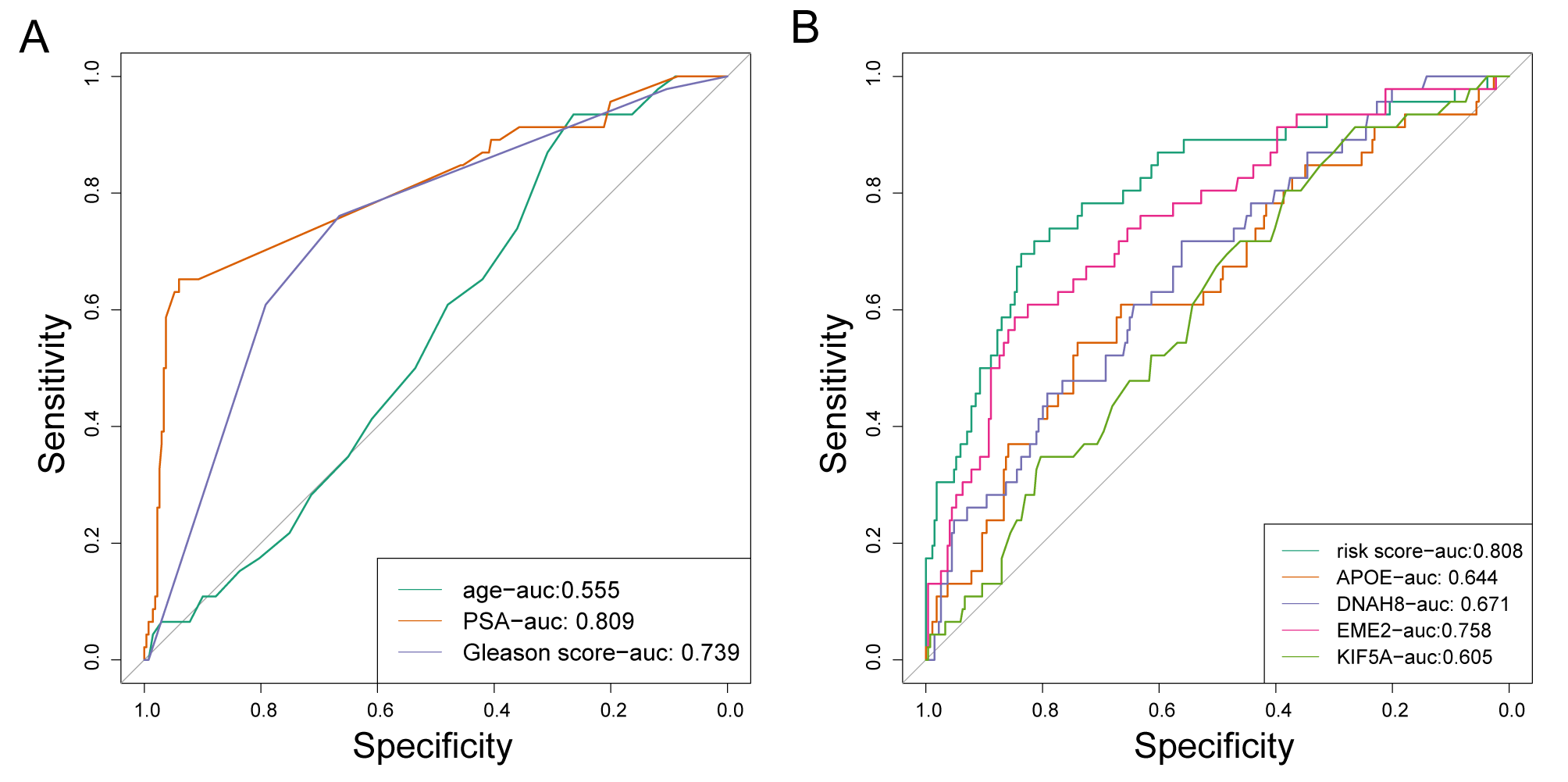

Supplement: Supplementary file 1 [file jcm-12-00654-s001.zip › Supplementary Figure 3.tif]
